# Dietary fiber sources affect reproductive and lactation outcomes and colostrum protein concentration in sows

**DOI:** 10.1007/s11250-026-05346-6

**Published:** 2026-09-17

**Authors:** Idael Matheus Góes Lopes, Marcelo Dourado de Lima, Soraia Viana Ferreira, Ana Clara Rodrigues de Oliveira, Betânia Rodrigues de Andrade, Rafaela Jorge Sarsur de Freitas Ribeiro, Naiara Cristina dos Santos Silveira, Artur Cavalcanti de Souza, Luisa Lopes da Rocha dos Santos, Luíza de Almeida Ramos, Francisco Carlos de Oliveira Silva, Eloísa de Oliveira Simões Saliba, Dalton de Oliveira Fontes

**Affiliations:** 1 Department of Animal Science, School of Veterinary Medicine, Federal University of Minas Gerais, Belo Horizonte, MG Brazil; 2 DanBred Brasil, Patos de Minas, MG Brazil; 3 Department of Nutrition and Animal Production, School of Veterinary Medicine and Animal Science, University of São Paulo, Pirassununga, SP Brazil; 4 Agricultural Research Institute of Minas Gerais, Viçosa, Minas Gerais Brazil; 5 Department of Animal Science, Federal University of Minas Gerais, Belo Horizonte, Minas Gerais 31270-901 Brazil

**Keywords:** Fiber fermentation, Sow nutrition, Parturition dynamics, Reproductive performance

## Abstract

This study aimed to evaluate the effects of supplementing different dietary fiber sources during late gestation. A total of 400 sows (parity 1–5; 215.7 ± 22.8 kg) were allocated in a randomized block design with four treatments: control (no added fiber), soybean hulls, lignocellulose, and micronized citrus pulp (*n* = 100 per treatment). Diets were isoenergetic and isonitrogenous, differing only in fiber source, and were provided from day 89 of gestation to weaning. Reproductive traits, piglet performance, fecal score, serum biochemical parameters, and colostrum yield and composition were evaluated. Blood metabolites were analyzed using an automatic biochemical analyzer, and glucose was measured with a portable glucometer. Statistical analyses included linear mixed-effects models, ANOVA with Tukey adjustment, Poisson and logistic regression models, and non-parametric tests when appropriate, with significance declared at *P* < 0.05. Fiber inclusion reduced stillbirth rates (*P* = 0.04), with lignocellulose showing the lowest incidence, and increased the number of weaned piglets (*P* = 0.01). Piglet weaning weight was higher in the micronized citrus pulp group (*P* = 0.04). Lactation mortality was reduced in fiber-fed sows compared to control (*P* < 0.01). Serum triglycerides were higher in sows fed lignocellulose (*P* = 0.04), while other biochemical parameters were unaffected. Colostrum protein concentration increased with micronized citrus pulp (*P* = 0.03), whereas fat, lactose, and colostrum yield were not influenced (*P* > 0.05). Dietary fiber supplementation during late gestation improved reproductive and lactation outcomes, particularly by reducing stillbirths and lactation mortality and enhancing piglet performance, while specific effects were observed on serum triglycerides and colostrum protein concentration.

## Introduction

Hyperprolific sows are capable of producing up to 20 live-born piglets per farrowing and weaning, on average, 33 to 35 piglets per sow per year (Johannsen et al. 2024). This improvement in reproductive performance has required increasingly precise dietary formulations to meet the rising nutritional demands associated with maintenance and production in these animals (Theil 2015). During late gestation, sows undergo marked physiological changes, including accelerated fetal growth, mammary gland development, colostrum synthesis, and maintenance of systemic metabolic functions (Theil 2015).

In this phase, mammary gland development is most pronounced, and the sow diet is a potential modulator of both colostrum yield and composition (Zhou et al. 2020). Evidence suggests that this effect is primarily related to colostrum fat content, which may be influenced by the inclusion of dietary fiber sources (Loisel et al. 2013; Feyera et al. 2019). This response has been attributed to increased production of short-chain fatty acids (SCFA), which are rapidly absorbed in the intestine and utilized as precursors for lipid synthesis.

In this context, nutritional strategies that support these physiological processes and contribute to more efficient farrowing kinetics are essential. Among such strategies, the inclusion of dietary fiber has shown positive effects on the reproductive and productive performance of sows (Williams et al. 2017; Van Hees et al. 2021; Li et al. 2021). Previous studies suggest that the beneficial effects of dietary fiber may involve mechanisms related to energy metabolism, intestinal barrier integrity, modulation of the gut microbiota, regulation of satiety and glycemia, and the production of short-chain fatty acids (SCFA), including acetate, propionate, and butyrate, which may contribute to changes in reproductive and physiological responses in sows (Feyera et al. 2017; Li et al. 2021).

Intestinal constipation, frequently observed during the peripartum period, is associated with prolonged farrowing duration, increased fetal hypoxia, and higher stillbirth rates. The inclusion of functional fibers in sow diets has proven to be an effective strategy to mitigate these effects (Priester et al. 2020). However, the choice of fiber source should consider its physicochemical characteristics, as solubility and fermentability are key determinants of its functionality within the gastrointestinal tract (Jonathan et al. 2012; Shang et al. 2021).

In Brazil, soybean hulls represent the main fibrous ingredient used in sow diets and are composed primarily of cellulose, hemicellulose, and lignin. This ingredient is predominantly insoluble and exhibits low fermentability. In contrast, by-products such as micronized citrus pulp have gained attention due to their high pectin content, soluble fiber fraction, and fermentable carbohydrates, in addition to low cost and high nutritional value (Ferreira et al. 2024). Currently, the market offers a wide range of fibrous ingredients, including both soluble and insoluble sources, as well as functional fibers with additional advantages, such as the absence of antinutritional factors and mycotoxins. Within this framework, the objective of the present study was to evaluate the effects of supplementing different dietary fiber sources in late-gestation sow diets on farrowing kinetics, reproductive performance, fecal score, serum biochemical parameters, colostrum yield and composition, and lactation performance.

## Materials and methods

### Animals, housing and experimental design

All experimental procedures were approved by the Animal Use Ethics Committee of the Federal University of Minas Gerais (UFMG; protocol no. 317/2022).

The experiment was conducted at a commercial farm located in the Alto Paranaíba region, Minas Gerais, Brazil (18°22′ S, 46°01′ W, 900 m a.s.l.), characterized by a tropical climate with a dry season, according to the Köppen–Geiger classification. Ambient temperature and relative humidity were continuously monitored throughout the experimental period using a data logger connected to a probe (GSP-8 Datalogger Elitech; accuracy ± 0.3 °C). Measurements were recorded at 60-s intervals. The equipment was positioned at the center of the barn, at animal height, allowing continuous assessment of environmental conditions. All facilities were equipped with evaporative cooling systems to ensure thermal comfort.

A total of 400 sows (Landrace females × Yorkshire boars; DanBred Brasil, Patos de Minas, MG, Brazil), with an average initial body weight of 215.7 ± 22.8 kg and parity ranging from 1 to 5, were assigned to four dietary treatments, with 100 sows per treatment, using a randomized design with restricted randomization. Initial body weight, backfat thickness, and parity order (parities 1, 2, 3, 4, and 5), recorded on day 89 of gestation, were used as balancing variables during treatment allocation to obtain comparable distributions among treatments. These variables did not define formal statistical blocks. The individual sow was considered the experimental unit. Sows remained on their respective experimental diets from day 89 of gestation until weaning (21 ± 2 d).

Sows were individually weighed at the beginning of the experiment (day 89 of gestation), during late gestation (day 110 of gestation), and at weaning using a digital scale (KM3-N; Coimma, São Paulo, Brazil). Backfat thickness and loin depth were measured at the P2 site, located 6.5 cm from the dorsal midline at the level of the last rib, on both sides of the body, using an ultrasound device (Mindray Ultrasound Scanner, DP2200).

During gestation, sows were housed in individual gestation stalls measuring 0.72 × 2.12 m, with partially slatted floors consisting of a solid front area (0.93 m) and a slatted rear area (1.05 m). At farrowing, sows were transferred to maternity facilities composed of rooms equipped with farrowing crates measuring 1.10 × 2.41 m. Each crate included an additional piglet creep area located at the front (1.53 × 0.53 m), fitted with heating devices to ensure adequate thermal conditions for neonatal piglets.

### Diets and feeding management

Four experimental diets were provided during the gestation period (Tables 1 and 2), whereas a standard lactation diet without the inclusion of additional fiber sources was offered during the lactation phase (Tables 3 and 4). The control diet was based on corn and soybean meal. The dietary treatments consisted of: (1) control diet without supplemental fiber; (2) diet supplemented with soybean hulls; (3) diet supplemented with lignocellulose; and (4) diet supplemented with micronized citrus pulp.

**Table 1:** Composition of the experimental diets

Ingredients	Treatments			
	Pre-weaning Control	Pre-weaning Soybean Hulls	Pre-weaning Lignocellulose	Pre-weaning Citrus Pulp
Ground corn	669.899	625.401	589.093	627.080
Soybean meal	260.000	255.000	273.000	261.000
Soybean hulls		40.000		
Lignocelulose			40.000	
Citrus Pulp (soluble fiber)				40.000
Sugar	25.000	25.000	25.000	25.000
Limestone	5.100	4.600	4.900	3.600
Dicalcium phosphate	15.000	15.000	15.000	15.000
Soybean oil		10.000	28.000	3.323
q.s. (fine ground corn)	2.171	1.889	2.513	2.047
Sodium bicarbonate	5.000	5.000	5.000	5.000
Kaolin	2.000	2.000	2.000	2.000
Salt	4.000	4.000	4.000	4.000
Probiotic	0.100	0.100	0.100	0.100
MOS (mannan-oligosaccharides)	0.800	0.800	0.800	0.800
Lacta 5 kg SUI (commercial product)	5.000	5.000	5.000	5.000
L-lysine	2.940	3.000	2.650	2.940
L-threonine	1.340	1.420	1.290	1.380
L-tryptophan	0.110	0.150	0.084	0.120
DL-methionine	1.540	1.640	1.570	1.610
Total (kg)	1.000	1.000	1.000	1.000

^a^Vitamin and mineral premix composition, provided per kg of gestation diet: iodine, 0.2 mg/kg; copper, 17 mg/kg; iron, 13 mg/kg; manganese, 16.7 mg/kg; selenium, 0.055 mg/kg; zinc, 18 mg/kg; cobalt, 0.03 mg/kg; pantothenic acid, 3.99 mg/kg; folic acid, 0.59 mg/kg; biotin, 0.16 mg/kg; niacin, 5.9 mg/kg; vitamin A, 2,390 IU/g; vitamin B1, 0.43 mg/kg; vitamin B2, 1.59 mg/kg; vitamin B6, 0.59 mg/kg; vitamin B12, 5.9 µg/kg; vitamin D3, 490 IU/g; vitamin E, 13 IU/kg; and vitamin K3, 0.49 mg/kg

**Table 2:** Calculated composition of the experimental diets

Parameters	Treatments			
	Pre-weaning Control	Pre-weaning Soybean Hulls	Pre-weaning Lignocellulose	Pre-weaning Citrus Pulp
Crude protein, %	17.500	17.516	17.500	17.500
Ether extract, %	2.391	3.371	4.895	2.680
Crude fiber, %	2.773	3.968	5.258	3.148
Ash, %	5.339	6.380	8.011	5.548
Available calcium, %	0.800	0.800	0.800	0.800
Total phosphorus, %	0.621	0.613	0.609	0.618
Available phosphorus, %	0.450	0.450	0.450	0.450
Total calcium, %	0.753	0.753	0.753	0.753
Metabolizable energy, kcal/kg	3224	3224	3224	3224
Net energy, Mcal/kg	2.414	2.405	2.430	2.403
Total valine, %	0.892	0.882	0.886	0.879
Digestible valine, %	0.736	0.726	0.737	0.728
Total lysine, %	1.122	1.132	1.121	1.120
Digestible lysine, %	1.000	1.000	1.000	1.000
Digestible Met + Cys, %	0.620	0.620	0.620	0.620
Digestible threonine, %	0.670	0.670	0.670	0.670
Digestible tryptophan, %	0.210	0.210	0.210	0.210
Chromium, mg/kg	-	-	-	-
Sodium, %	0.316	0.315	0.315	0.316
Chloride, %	0.305	0.301	0.299	0.302
Iron, mg/kg	67.200	67.200	67.200	67.200
Copper, mg/kg	87.500	87.500	87.500	87.500
Manganese, mg/kg	33.600	33.600	33.600	33.600
Zinc, mg/kg	92.400	92.400	92.400	92.400
Cobalt, mg/kg	0.168	0.168	0.168	0.168
Iodine, mg/kg	1.168	1.168	1.168	1.168
Selenium, mg/kg	0.294	0.294	0.294	0.294
Vitamin A, IU/g	12.000	12.000	12.000	12.000
Vitamin D3 (cholecalciferol), IU/g	2.500	2.500	2.500	2.500
Vitamin E, IU/g	85.517	84.516	83.775	84.586
Vitamin K3, mg/kg	2.500	2.500	2.500	2.500
Vitamin B1 (thiamine), mg/kg	4.384	4.259	4.196	4.274
Vitamin B2 (riboflavin), mg/kg	8.000	8.000	8.000	8.000
Vitamin B6 (pyridoxine), mg/kg	3.000	3.000	3.000	3.000
Vitamin B12, µg/kg	30.000	30.000	30.000	30.000
Niacin, mg/kg	30.000	30.000	30.000	30.000
Pantothenic acid, mg/kg	20.000	20.000	20.000	20.000
Folic acid, mg/kg	3.000	3.000	3.000	3.000
Biotin, mg/kg	0.820	0.820	0.820	0.820
Choline, g/kg	1.801	1.738	1.749	1.760
Vitamin C, mg/kg	-	-	-	-
Total micro-premix, %	2.500	2.500	2.500	2.500
Acid detergent fiber (ADF), %	4.248	5.909	6.902	4.643
Neutral detergent fiber (NDF), %	12.878	14.512	11.950	13.040

**Table 3:** Centesimal composition of the diet used during the lactation phase

Lactation Diet	
Ingredients	Inclusion (%)
Ground corn	500.82
Soybean meal	317.33
Promil	25.33
Meat and bone meal	30.00
Sugar	40.00
Degummed soybean oil	45.33
q.s. (fine ground corn)	5.47
Dicalcium phosphate	12.47
Sodium bicarbonate	1.33
Kaolin	2.00
Palatability enhancer	0.10
Enzymes	0.05
Common salt	5.00
Citric acid	1.47
A.M.A. Biosyn	0.15
Chromium	0.10
Selenium	0.06
Diatomite	0.50
L-carnitine	0.02
Vitamin–mineral premix^1^	5.00
L-lysine	2.50
L-threonine	1.66
L-tryptophan	0.50
L-valine	0.80
DL-methionine	1.61
Flavoring agent	0.40
Total	1.000

^1^Vitamin and mineral premix composition, provided per kg of lactation diet: iodine, 0.23 mg/kg; copper, 75 mg/kg; iron, 15 mg/kg; manganese, 7.9 mg/kg; selenium, 0.065 mg/kg; zinc, 33 mg/kg; cobalt, 0.038 mg/kg; pantothenic acid, 4.99 mg/kg; folic acid, 0.67 mg/kg; biotin, 0.165 mg/kg; niacin, 6.74 mg/kg; vitamin A, 2,690 IU/g; vitamin B1, 0.49 mg/kg; vitamin B2, 1.79 mg/kg; vitamin B6, 0.67 mg/kg; vitamin B12, 6.74 µg/kg; vitamin D3, 560 IU/g; vitamin E, 15.74 IU/kg; and vitamin K3, 0.56 mg/kg

**Table 4:** Calculated composition of the diet used during the lactation phase

Nutrient	Unit	Calculated value
Crude protein	%	20.92
Ether extract	%	6.88
Crude fiber	%	2.97
Ash	%	10.15
Available calcium	%	0.90
Total calcium	%	0.85
Total phosphorus	%	0.75
Available phosphorus	%	0.56
Metabolizable energy for pigs	Kcal/kg	3.50
Net energy for pigs	Mcal/kg	2.61
Total valine	%	1.11
Digestible valine	%	0.95
Total lysine	%	1.30
Digestible lysine for pigs	%	1.17
Digestible Met + Cys for pigs	%	0.70
Digestible threonine for pigs	%	0.82
Digestible tryptophan for pigs	%	0.28
Chromium	mg/kg	0.40
Sodium	%	0.28
Iron	mg/kg	67.20
Copper	mg/kg	87.50
Manganese	mg/kg	33.60
Zinc	mg/kg	92.40
Cobalt	mg/kg	0.17
Iodine	mg/kg	1.21
Selenium	mg/kg	0.35
Vitamin A	IU/g	12.00
Vitamin D3 (cholecalciferol)	IU/g	2.50
Vitamin E	IU/g	70.00
Vitamin K3	mg/kg	2.50
Vitamin B1 (thiamine)	mg/kg	2.20
Vitamin B2 (riboflavin)	mg/kg	8.00
Vitamin B6 (pyridoxine)	mg/kg	3.00
Vitamin B12	µg/kg	30.00
Niacin	mg/kg	30.00
Pantothenic acid	mg/kg	20.00
Folic acid	mg/kg	3.00
Biotin	mg/kg	0.82
Choline	g/kg	1.89

The inclusion levels of micronized citrus pulp and lignocellulose were established according to the manufacturers’ recommendations, with formulation adjustments to meet the nutritional requirements proposed by Rostagno et al. (2017). The supplemental fiber ingredients were included at the same dietary inclusion rate (40 g/kg), rather than being formulated to provide equivalent amounts of dietary fiber. Consequently, adjustments in the proportions of other dietary ingredients, including soybean oil, were required to maintain the gestation diets as isoenergetic and isonitrogenous, resulting in differences in fiber and ether extract concentrations among treatments (Tables 1 and 2). The lower NDF concentration observed in the lignocellulose diet, despite its higher ADF and crude fiber concentrations, reflects differences in fiber fraction composition and the ingredient substitutions required during diet formulation.

During gestation, sows were fed once daily at 07:00 h. From day 110 of gestation onwards, sows were transferred to the farrowing facility, and the diet was portioned/subdivided into three daily meals, provided at 07:00 h, 13:00 h, and 15:30 h. Feed allowance during gestation and until farrowing was maintained at 3.0 kg per sow per day until farrowing. Water was available ad libitum throughout the experimental period.

### Blood sampling and biochemical analyses

During gestation, blood samples were collected on days 89 and 110 of gestation, prior to the morning feeding, by jugular venipuncture using 40 × 1.2 mm needles. Samples were collected into two types of tubes, with and without sodium fluoride. Tubes containing sodium fluoride were centrifuged within 20 min after collection to obtain plasma. Samples collected in tubes without additives were kept at room temperature for 2 h prior to centrifugation to allow clot formation and serum separation.

Both plasma and serum samples were centrifuged at 1,500 × g for 15 min at room temperature using a bench centrifuge (80-2B; Centribio). The resulting plasma and serum were stored at − 20 °C until further analyses.

Plasma and serum concentrations of total protein, alanine aminotransferase, aspartate aminotransferase, triglycerides, high-density lipoproteins, low-density lipoproteins, alkaline phosphatase, lactate, cholesterol, and creatinine were determined using an automatic biochemical analyzer (COBAS MIRA Plus; Roche Diagnostics International Ltd., Rotkreuz, Switzerland) at the Multilab-HV/UFMG laboratory, with commercial kits (Biotécnica^®^, MG, Brazil).

### Blood glucose measurement

Blood glucose concentrations were assessed using a portable glucometer (Accu-Chek Guide Meter™, Roche Diabetes Care, Inc., Indianapolis, IN, USA.), as previously described by Carnevale et al. (2023). Measurements were performed at the onset of farrowing and immediately after the birth of the last piglet. The final glucose value for each sow corresponded to the measurement obtained at the completion of farrowing.

### Piglet management and reproductive measurements

During farrowing, the following variables were recorded: total number of piglets born, number of live-born piglets, stillborn piglets, and mummified fetuses, as well as the birth time and individual birth weight of each piglet. Neonatal management procedures included umbilical cord ligation, with cutting performed 3 to 5 cm from the insertion point, followed by disinfection with a 5% iodine solution. All piglets were stimulated to ingest colostrum immediately after birth.

Litters were equalized within each dietary treatment, maintaining two piglets more than the number of functional teats of each sow. From seven days of age onward, piglets had access to creep feed. At three days of age, piglets received an intramuscular iron injection to prevent iron-deficiency anemia, and tail docking was performed. In addition, an oral energy supplement was administered, consisting of a probiotic blend containing *Saccharomyces cerevisiae*,* Bifidobacterium bifidum*,* Streptococcus thermophilus*,* Lactobacillus acidophilus*,* and Bacillus amyloliquefaciens.*

Placental efficiency was calculated as the ratio between placental weight, measured after complete expulsion, and total litter weight, according to Van Rens et al. (2005). Farrowing duration (min) and birth interval (min) were also determined based on the exact time of birth of each piglet.

During lactation, piglet losses and mortality were recorded daily for each sow. The variable “removed piglets (%)” included piglets with signs of low viability (runts), as well as those removed due to disease or injury. At weaning, litters were weighed using a digital scale (KM3-N; Coimma, São Paulo, Brazil) to determine piglet weaning weight and weight gain during lactation.

### Fecal score assessment

Intestinal activity was monitored in 50 sows per treatment for 20 consecutive days prior to farrowing through daily qualitative fecal evaluations. Each morning, before facility cleaning, feces were visually assessed and classified according to the scoring system proposed by Oliviero et al. (2009).

The fecal score scale ranged from 0 to 5, where 0 indicated absence of feces; 1 indicated dry, pellet-like feces; 2 indicated feces with a consistency between dry and normal; 3 indicated normal feces, soft, firm, and well-formed; 4 indicated feces between normal and wet, still formed but lacking firmness; and 5 indicated very wet, unformed feces with a liquid appearance.

### Colostrum collection and composition analysis

Colostrum analyses were performed at the Milk Technology Laboratory of the Federal University of Minas Gerais (UFMG). In the present study, colostrum samples were collected from 18 sows per treatment to determine protein (%), fat (%), and lactose (%) concentrations. Colostrum was manually collected by hand milking immediately after the birth of the first piglet, sampling all functional teats of each sow. Subsequently, a pooled sample of 50 mL of colostrum per sow was obtained and immediately stored at − 20 °C until analysis. Prior to laboratory determinations, samples were thawed and allowed to reach room temperature.

### Colostrum yield estimation

Colostrum yield was estimated in 18 sows (litters) per treatment, corresponding to the same subsample used for colostrum composition analysis, according to the methodology described by Devillers et al. (2004). Colostrum production was calculated as the sum of the individual colostrum intake of each piglet within the litter (SCI). The estimation was based on the following equation:

$$\eqalign{ {\rm{SCI = }} & - {\rm{217}},{\rm{4}}{\mkern 1mu} {\rm{ + }}{\mkern 1mu} {\rm{0}},{\rm{217*t + 1861019*}}{{{\rm{BW24h}}} \over {\rm{t}}} \cr & {\rm{ + BW0h*}}\left( {{\rm{54}},{\rm{80}} - {\rm{1861019}}} \right){\rm{/t}}){\rm{*}}({\rm{0}},{\rm{9985}} \cr & - {\rm{3}},{\rm{7*10 - 4}} \times {\rm{tfs + 6}},{\rm{1*10 - 7*tfs}}) \cr}$$

Where BW₀h is the body weight of each piglet at birth; BW₂₄h is the body weight of the piglet 24 h after birth; and t is the time interval between the first and second weighing, fixed at 1,440 min (24 h × 60 min). The time from birth to first colostrum intake (tfs) was not individually recorded and was therefore standardized at 30 min for all piglets for the purpose of the estimation. This assumption should be considered when interpreting colostrum yield estimates, as individual variation in the time to first colostrum intake was not captured by the calculation.

### Weaning-to-estrus interval assessment

After weaning, sows were returned to the gestation facility, where they remained under a flushing management protocol until estrus detection. During this period, a mature boar was moved through the barn by trained personnel, allowing direct nose-to-nose contact with the sows to stimulate estrus expression.

Estrus was identified based on the presence of a positive standing reflex in response to back-pressure applied by the operator in the presence of the boar. The weaning-to-estrus interval was defined as the number of days from weaning until the first observed estrus.

### Statistical analysis

Data meeting parametric assumptions were analyzed using a linear mixed-effects model fitted with the lmer function of the lme4 package in R software (R Core Team 2023). Initial body weight and backfat thickness were used only as balancing variables during treatment allocation and were not included as covariates in the inferential model because they did not define formal statistical blocks. Treatment and parity order were included as fixed effects, whereas the week in which the sows entered the experiment was included as a random effect, according to the following structure:

$${\rm{}}{{\rm{Y}}_{{\rm{ijk}}}}{\rm{ = }}\mu {\rm{ + Treatmen}}{{\rm{t}}_{\rm{i}}}{\rm{ + Parit}}{{\rm{y}}_{\rm{j}}}{\rm{ + }}\left( {{\rm{1|Week}}} \right){\rm{ + }}{\varepsilon _{{\rm{ijk}}}}$$

Where: Yijk is the response variable, is the overall mean, Treatmenti is the fixed effect of dietary fiber source, Parityj is the fixed effect of parity order, Weekk is the random effect associated with the week in which sows entered the study at day 89 of gestation, and ijk is the residual error, assumed to be normally distributed as N (0, σ²).

The significance of fixed effects and their interactions was assessed using analysis of variance (ANOVA) based on the fitted mixed model. When significant effects were detected, adjusted (marginal) means were estimated using the emmeans function of the emmeans package, and pairwise comparisons were performed using Tukey’s test with appropriate adjustment for multiple comparisons.

To evaluate the effects of dietary fiber sources on the weaning-to-estrus interval, stillborn piglets, and mummified fetuses, a Poisson regression model was applied. Farrowing rate and anestrus rate at the subsequent reproductive cycle were analyzed using logistic regression models.

Fecal score data were initially screened for outliers using Grubbs’ test to exclude extreme observations that could bias the results. After outlier removal, data were tested for normality, which indicated a non-normal distribution. Therefore, group comparisons were performed using the Kruskal–Wallis test. When significant differences were detected, Dunn’s test was applied as a post hoc procedure to identify statistically significant differences among treatments.

Colostrum yield data were evaluated for normality using the Shapiro–Wilk test and for homogeneity of variances using Levene’s test, and subsequently analyzed by analysis of variance (ANOVA), considering blocks, using the Glimmix procedure of SAS software (Statistical Analysis System, version 9). Results were considered statistically significant when *P* < 0.05.

Given the number of response variables evaluated across reproductive, lactation, biochemical, fecal, and colostrum outcomes, statistical tests were interpreted individually at the predefined significance level of *P* < 0.05, without adjustment for multiplicity across endpoints. Therefore, findings with P-values close to the significance threshold should be interpreted cautiously in view of the potential for type I error arising from multiple testing.

## Results

### Ambient temperature and relative humidity

During the gestation period, ambient temperature ranged from a minimum of 17.8 °C to a maximum of 26.4 °C, with an average of 20.4 °C. Relative humidity (RH) varied between 58.2% and 92.4%, corresponding to the temperature fluctuations described above, with an average value of 79.9%. During lactation, ambient temperature ranged from 17.8 °C to 31.3 °C, with a mean of 21.7 °C. Relative humidity ranged from 38.0% to 90.6%, resulting in an average RH of 74.3%.

### Body condition

The body condition of sows during gestation and lactation fed different dietary fiber sources is presented in Table 5. Confirming the effectiveness of the randomization procedure applied at day 89 of gestation, no effects of dietary fiber sources were observed (*P* > 0.05) on initial body weight, initial backfat thickness (BF), or initial loin depth (LD).

**Table 5:** Performance of sows during gestation and lactation fed different fiber sources

Parameters	Treatments				SEM	*P*-value
	Control	Soybean hulls	Lignocellulose	Micronized citrus pulp		
Initial body weight, kg	215.56	215.35	215.05	215.26	1.11	0.99
Pre-farrowing body weight, kg	241.57	240.18	241.25	239.34	1.21	0.81
Weaning body weight, kg	217.27	216.02	215.57	211.89	1.3	0.31
Initial backfat thickness, mm	16.26	16.08	15.29	15.24	0.2	0.79
Pre-farrowing backfat thickness, mm	16.56	16.83	15.74	16.49	0.19	0.27
Weaning backfat thickness, mm	13.4	12.88	12.47	12.81	0.15	0.28
Backfat change (pre-farrowing − weaning), mm	-3.54	-3.85	-3.30	-3.61	0.16	0.71
Initial loin depth, mm	44.3	43.68	43.79	44.46	0.29	0.76
Pre-farrowing loin depth, mm	43.65	44.14	43.86	43.76	0.28	0.93
Weaning loin depth, mm	42.59 a	40.28 b	40.61 ab	40.14 b	0.27	0.02
Loin depth change (pre-farrowing − weaning), mm	3.06 b	3.86 a	3.25 ab	3.62 a	0.31	0.05

Within a row, means followed by the same letter (or no letter) do not differ significantly (*P* > 0.05) according to Tukey’s test. kg = kilogram; mm = millimeter; SEM = standard error of the mean

Similarly, no effects (*P* > 0.05) of dietary fiber sources were detected on pre-farrowing body weight or body weight at weaning. Backfat thickness measured at the different stages (initial, pre-farrowing, and weaning), as well as changes in BF over time, were not influenced by dietary treatments (*P* > 0.05).

However, a significant effect was observed for loin depth at weaning (*P* = 0.02). Sows fed the control diet exhibited greater loin depth at weaning compared with those fed soybean hulls and micronized citrus pulp, whereas sows fed lignocellulose showed intermediate values, not differing statistically from the other treatments. In addition, the change in loin depth from pre-farrowing to weaning was significant (*P* = 0.05). The control group exhibited the smallest reduction in loin depth, whereas sows fed soybean hulls showed the greatest losses, those fed micronized citrus pulp also exhibited pronounced reductions, and sows fed lignocellulose presented intermediate loin depth losses during lactation.

### Reproductive performance

No differences (*P* > 0.05) were observed among dietary treatments for total piglets born, live-born piglets, mummified fetuses, placental efficiency, farrowing duration, birth interval, farrowing rate, or initial and final blood glucose concentrations, as shown in Table 6.

**Table 6:** Reproductive performance of sows fed different fiber sources

Parameters	Treatments				SEM	*P*-value
	Control	Soybean hulls	Lignocellulose	Micronized citrus pulp		
Total born	17.62	16.64	17.01	16.90	0.15	0.16
Born alive	16.25	15.48	16.01	15.80	0.16	0.24
Stillborn	0.897 a	0.658 b	0.581 c	0.620 b	0.06	0.04
Mummified	0.48	0.51	0.40	0.46	0.05	0.67
Placental weight, kg	4.02 b	4.01 b	4.35 a	4.06 b	0.05	0.04
Placental efficiency, %	5.39	5.28	5.21	5.23	0.03	0.38
Farrowing duration, min	322.61	332.15	315.35	329.09	6.34	0.63
Birth interval, min	18.31	19.96	18.54	19.47	0.37	0.39
Farrowing rate, %	98.00	92.07	96.00	96.03	0.01	0.22
Initial glucose, mg/dL	72.57	71.22	70.84	69.83	0.62	0.51
Final glucose, mg/dL	76.46	78.32	80.38	79.07	0.59	0.20

Within a row, means followed by the same letter (or no letter) do not differ significantly (*P* > 0.05) according to Tukey’s test. kg = kilogram; mg = milligram; dL = deciliter; min = minutes;

^1^SEM = standard error of the mean

In contrast, the inclusion of functional dietary fibers reduced the incidence of stillborn piglets (*P* = 0.04). Sows fed lignocellulose exhibited the lowest stillbirth incidence, followed by those fed micronized citrus pulp and soybean hulls, whereas the control group showed the highest number of stillborn piglets. In addition, placental weight was greater (*P* = 0.04) in sows fed lignocellulose compared with those fed the control, soybean hull diets, and micronized citrus pulp.

### Litter and sow performance

Litter performance results are presented in Table 7. No differences (*P* > 0.05) were observed among dietary treatments for birth weight, coefficient of variation (CV) of birth weight, number of piglets after litter equalization, average piglet weight after equalization, number of removed piglets, weaning-to-estrus interval, or anestrus rate at the subsequent farrowing.

**Table 7:** Litter and sow performance as affected by different fiber sources

Parameters	Treatments				SEM	*P*-value
	Control	Soybean hulls	Lignocellulose	Micronized citrus pulp		
Birth weight, kg	1.288	1.339	1.336	1.303	0.01	0.21
Birth weight CV^1^, %	24.95	24.01	23.59	24.88	0.44	0.57
Piglets standardized, n	16.42	16.41	16.25	16.28	0.03	0.21
Mean weight after standardization, kg	1.403	1.404	1.423	1.405	0.01	0.95
Weaned piglets, n	14.42 b	14.79 ab	14.91 a	14.56 ab	0.05	0.01
Mean weaning weight, kg	5.745 b	6.021 ab	5.959 ab	6.133 a	0.05	0.04
Lactation loss, %	2.00 a	1.62 b	1.34 b	1.72 b	0.04	< 0.01
Lactation mortality, %	10.04 a	8.47 ab	7.07 b	7.74 b	0.35	< 0.01
Removed piglets, %	2.13	1.40	1.23	2.83	0.20	0.42
Weaning-to-estrus interval (next parity), d.	4.89	5.12	5.09	5.20	0.04	0.29
Anestrus rate in the subsequent parity, %	3.72	3.88	3.90	3.88	0.13	0.40

Within a row, means followed by the same letter (or no letter) do not differ significantly (*P* > 0.05) according to Tukey’s test. kg = kilogram; d = days

^1^CV = coefficient of variation;

^2^SEM = standard error of the mean;

However, the number of weaned piglets was greater (*P* = 0.01) in sows fed lignocellulose compared with those fed the control diet. Sows fed soybean hulls and micronized citrus pulp showed intermediate values and did not differ statistically from the other treatments.

Mean piglet weaning weight was also affected by dietary treatments (*P* = 0.04). Piglets from sows fed micronized citrus pulp showed the highest values, those from sows fed soybean hulls and lignocellulose were intermediate, and piglets from control sows had the lowest weights.

Piglet losses during lactation were significantly affected by dietary treatments (*P* < 0.01); sows fed lignocellulose, soybean hulls, and micronized citrus pulp showed similar losses, all of which were lower than those observed in the control group. Similarly, lactation mortality differed among treatments (*P* < 0.01). Sows fed lignocellulose and micronized citrus pulp exhibited the lowest mortality rates, those fed soybean hulls showed intermediate values, whereas the control group recorded the highest mortality rate.

### Serum biochemical parameters

The results of serum biochemical parameters evaluated before and after the dietary treatments are presented in Table 8. During the pre-treatment period, no differences (*P* > 0.05) were observed among treatments for serum concentrations of creatinine, lactate, triglycerides, total protein, cholesterol, urea, alanine aminotransferase, aspartate aminotransferase, high-density lipoproteins, low-density lipoproteins, or alkaline phosphatase.

**Table 8:** Serum or plasma biochemical parameters of sows at days 90 and 112 of gestation fed different fiber sources

Parameters	Treatments				SEM	*P*-value
	Control	Soybean hulls	Lignocellulose	Micronized citrus pulp		
Pretreatment						
Total protein (g/L)	6.20	6.21	6.38	6.02	0.06	0.21
ALT (U/L)	27.29	26.10	25.22	25.44	0.55	0.47
AST (U/L)	10.84	10.90	10.27	11.98	0.63	0.81
Triglycerides (mg/L)	48.51	56.23	50.50	54.64	2.41	0.66
HDL (mg/dL)	26.79	25.83	22.00	22.82	0.73	0.08
LDL (mg/dL)	33.24	32.01	27.79	30.20	1.07	0.24
Alkaline phosphatase (U/L)	95.58	71.51	89.79	73.47	4.62	0.12
Lactate (mmol/L)	50.15	43.09	37.40	43.80	1.75	0.09
Cholesterol (mg/dL)	68.38	69.72	56.04	61.50	2.60	0.21
Urea (mmol/L)	30.69	29.69	25.32	27.21	0.94	0.19
Creatinine (µmol/L)	2.31	2.25	2.41	2.30	0.06	0.82
**Post-Treatment**						
Total protein (g/L)	5.99	5.45	5.80	5.47	0.09	0.10
ALT (U/L)	26.41	27.71	30.35	26.02	1.09	0.50
AST (U/L)	11.35	10.53	12.35	11.48	0.38	0.49
Triglycerides (mg/L)	77.34 b	85.73 ab	92.97 a	88.70 ab	2.01	0.04
HDL (mg/dL)	30.37	32.18	31.11	29.38	0.62	0.47
LDL (mg/dL)	47.45	45.11	49.68	42.33	1.67	0.47
Alkaline phosphatase (U/L)	47.06	45.50	49.63	44.01	1.94	0.78
Lactate (mmol/L)	26.70	28.95	27.34	24.05	0.81	0.22
Cholesterol (mg/dL)	81.87	77.68	86.05	76.52	1.55	0.15
Urea (mmol/L)	33.27	34.14	36.66	34.27	0.92	0.62
Creatinine (µmol/L)	2.50	2.52	2.57	2.51	0.04	0.96

Within a row, means followed by the same letter (or no letter) do not differ significantly (*P* > 0.05) according to Tukey’s test. ALT = alanine aminotransferase; AST = aspartate aminotransferase; HDL = high-density lipoproteins; LDL = low-density lipoproteins;

^1^ SEM = standard error of the mean

In the post-treatment period, only serum triglyceride concentration differed among treatments (*P* = 0.04). Sows fed lignocellulose exhibited higher triglyceride concentrations compared with the control group, whereas sows fed soybean hulls and micronized citrus pulp showed intermediate values and did not differ statistically from the other treatments.

### Fecal score

Over the total period, fecal score differed among dietary treatments (*P* < 0.01). Sows fed lignocellulose and micronized citrus pulp exhibited the highest fecal scores, followed by those fed soybean hulls, whereas the control group showed the lowest value (Table 9).

**Table 9:** Impact of dietary fiber levels on faecal score of gestating sows

Parameters	Treatments				SEM	*P*-value
	Control	Soybean hulls	Lignocellulose	Micronized citrus pulp		
Total period	0.189b	0.635c	0.855a	0.800a	0.08	< 0.01

Treatments followed by the same letter do not differ from each other at the 5% probability level according to Dunn’s test

^1^SEM = standard error of the mean

### Colostrum composition

The results of colostrum composition are presented in Table 10. No significant effects of dietary fiber sources were observed on colostrum fat concentration (*P* = 0.57). Similarly, lactose concentration did not differ among treatments (*P* = 0.80). In contrast, dietary treatments significantly affected colostrum protein concentration (*P* = 0.03). Sows fed micronized citrus pulp exhibited a higher colostrum protein content compared with those fed the other dietary treatments.

**Table 10:** Effect of dietary fiber levels on colostrum composition of gestating sows

Parameters	Treatments				SEM	*P*-value
	Control	Soybean hulls	Lignocellulose	Micronized citrus pulp		
Fat (%)	5.60	6.29	5.73	6.30	0.21	0.57
Protein (%)	12.08 b	11.85 b	12.27 b	15.69 a	0.49	0.03
Lactose (%)	4.65	5.45	5.31	5.30	0.26	0.80

Within a row, means followed by the same letter (or no letter) do not differ significantly (*P* > 0.05) according to Tukey’s test

^1^SEM = standard error of the mean

### Colostrum yield

Colostrum yield results are presented in Table 11. According to the statistical analysis, no differences (*P* = 0.234) were observed among dietary treatments with different fiber sources.

**Table 11:** Effect of dietary fiber levels on colostrum yield of sows

Parameters	Treatments				SEM	*P*-value
	Control	Soybean hulls	Lignocellulose	Micronized citrus pulp		
Colostrum yield (kg)	3.986	4.428	3.616	3.561	0.17	0.234

^1^SEM = standard error of the mean

## Discussion

Currently, dietary fiber sources available for sow diet formulation exhibit wide variability in origin, inclusion levels, and physicochemical properties. In Brazil, soybean hulls and wheat bran are commonly used in sow diets, which partially explains the heterogeneity of results reported in the literature when alternative fiber sources are evaluated (Ferreira et al. 2024). This variability reinforces the importance of comparative studies under controlled field conditions, such as the present work, to better understand the biological responses associated with different fiber types.

The inclusion of functional dietary fibers in sow diets has been consistently associated with several benefits, including improved body condition maintenance, reduced constipation, lower stillbirth rates, and increased satiety (Sun et al. 2015; Li et al. 2021). In addition, fiber supplementation during gestation has been reported to favor voluntary feed intake during lactation, contributing to improved performance of both sows and their litters. These responses are particularly relevant in modern hyperprolific genotypes, in which metabolic demands during late gestation and early lactation are markedly increased.

The mechanisms underlying the effects of gestational fiber intake on lactation performance are not yet fully elucidated (Odakura et al. 2023). However, gastric distension and increased gastrointestinal tract capacity have been proposed as key factors contributing to greater feed intake during lactation (Darroch et al. 2008). Hormonal regulation also appears to play an important role. Quesnel et al. (2009) reported that increased fiber intake during gestation reduced circulating leptin concentrations, a hormone closely involved in appetite regulation, potentially facilitating higher feed intake postpartum.

In the present study, no differences were observed among treatments for pre-farrowing body weight or body weight at weaning, which is consistent with previous reports showing limited effects of fiber source or inclusion level on sow body weight (Li et al. 2021; Ferreira et al. 2024; Oh et al., 2024). Nevertheless, Shang et al. (2021) demonstrated that the inclusion of 20% beet pulp improved body condition maintenance by reducing body weight loss during lactation. Although body weight did not differ among treatments in the current study, changes in body tissue reserves were evident.

Backfat thickness was not affected by dietary treatments at pre-farrowing or weaning, corroborating the findings of Zhou et al. (2020), who also reported no effect of dietary fiber levels on backfat thickness when fed from late gestation to farrowing. Conversely, loin depth at weaning was significantly affected by dietary fiber source. Mobilization of body reserves during lactation is a common physiological response to sustain milk production, often resulting in muscle tissue loss (Laws et al. 2009). In agreement with our findings, Renteria-Flores et al. (2008) reported reduced loss of body reserves in sows fed fiber-rich diets compared with those fed control diets, highlighting the role of dietary fiber in modulating tissue mobilization during lactation.

The number of total and live-born piglets was not influenced by dietary fiber source in the present study. However, positive effects of dietary fiber on these parameters have been reported when wheat bran (Che et al. 2011), wheat straw (Veum et al. 2009), or beet pulp (Van der Peet-Schwering et al., 2003) were included in sow diets. Such discrepancies among studies may be attributed to differences in fiber source, inclusion level, and, most importantly, the proportion of soluble and insoluble fiber fractions, which directly influence digestive physiology and reproductive performance (Li et al. 2019a, b).

In contrast, the absence of fiber in the control diet negatively affected stillbirth rate, reinforcing the importance of fiber inclusion during late gestation. Dietary fiber reduces constipation, a condition strongly associated with prolonged farrowing, fetal hypoxia, and increased stillbirth incidence. In the present study, all fiber-containing diets reduced stillbirth rates compared with the control, highlighting the functional relevance of fiber supplementation.

The stillbirth response observed in the present study differs from that reported by Ferreira et al. (2024), who evaluated 220 sows receiving a control diet or diets containing soybean hulls, micronized citrus pulp, or coffee husks. In that earlier independent trial, sows fed soybean hulls had a higher stillbirth incidence than control sows, whereas micronized citrus pulp and coffee husks produced intermediate responses. In the present study, all fiber-containing diets reduced stillbirth incidence compared with the fiber-free control, with lignocellulose producing the lowest value. The sample size was increased from 220 to 400 sows to provide more precise estimates of reproductive and lactation outcomes characterized by substantial between-sow variability. The control, soybean hull, and micronized citrus pulp treatments were retained as common references, whereas coffee husks were replaced with lignocellulose to extend the evaluation to a functional, predominantly insoluble fiber source. Direct comparisons should nevertheless be made cautiously because the experiments also differed in the period of dietary exposure and the inclusion rates of the fiber sources. Collectively, the contrasting findings reinforce that reproductive responses to dietary fiber depend on the specific source, inclusion rate, and period of supplementation.

Placental weight was greater in sows fed lignocellulose compared with the other dietary treatments, although this response was not accompanied by differences in placental efficiency or piglet birth weight. Placental growth and function are closely associated with nutrient transfer capacity and fetal development, and changes in placental size may represent adaptive responses to the intrauterine environment (Vonnahme and Lemley 2012). In this context, the greater placental weight observed with lignocellulose, without a corresponding increase in fetal growth, may reflect compensatory placental growth rather than enhanced placental efficiency. However, because placental efficiency did not differ among treatments, the present results do not provide evidence of impaired placental function, and the biological significance of the increased placental weight should therefore be interpreted with caution.

Constipation during late gestation is a common condition in sows, affecting up to 64.6% of animals with severe symptoms (Pearodwong et al. 2016). This condition is exacerbated by restricted feeding, reduced physical activity, and increased uterine volume during late gestation (Tabeling et al. 2003; Lu et al. 2022). Oliviero et al. (2009) demonstrated that constipated sows experienced prolonged farrowing durations exceeding 300 min and higher stillbirth rates.

In this context, lignocellulose emerged as a particularly effective insoluble fiber source. Insoluble fibers increase fecal bulk by enhancing water retention and fecal mass, thereby improving fecal consistency and reducing constipation (Oliviero et al. 2010). In the present study, higher fecal scores, indicating reduced severity of constipation, coincided with lower stillbirth rates, particularly in sows fed lignocellulose and micronized citrus pulp. These findings suggest that improved fecal consistency may be associated with the reproductive responses observed in fiber-fed sows.

Similarly, Krogh et al. (2015) observed a higher frequency of soft feces (score ≥ 3) in sows fed beet pulp, a predominantly soluble fiber source, compared with low-fiber diets. In the present study, although fecal scores remained below the value corresponding to normal fecal consistency across all treatments, the higher scores observed in fiber-fed sows indicate a reduced severity of constipation compared with the control group rather than an absence of digestive disturbance.

Improved intestinal transit associated with fiber intake facilitates the elimination of waste products and reduces gastrointestinal discomfort, which may indirectly reduce farrowing complications (Yu et al. 2021). Despite these benefits, no effects were observed on farrowing duration or birth interval in the present study. Although insoluble fibers stimulate peristalsis and reduce intestinal transit time (Wenk 2001; Low et al. 2020), their impact on farrowing kinetics appears to be influenced by additional factors, including fiber fermentability and metabolic responses.

Short-chain fatty acids (SCFA), produced through colonic fermentation of dietary fiber, have been proposed as potential mediators of the effects of fiber on parturition through their contribution to energy metabolism and smooth muscle activity (Bugaut 1987; Le Blanc et al. 2017; Tian et al. 2020). However, SCFA concentrations and gut microbiota composition were not measured in the present study; therefore, their involvement in the responses observed here remains hypothetical. Moreover, no effects on farrowing duration or birth interval were observed. In contrast, Oh et al. (2022) reported shorter farrowing duration in sows fed higher-fiber diets, suggesting that responses may depend on fiber dose and source.

Regarding serum biochemical parameters, no differences were observed before dietary treatments, confirming baseline homogeneity among groups. After treatment, only triglyceride concentrations differed, with higher values observed in sows fed lignocellulose. Serum triglyceride concentrations reflect the balance among intestinal lipid absorption, hepatic synthesis and secretion, and peripheral tissue uptake (Campbell 2004), and dietary fiber may also influence lipid metabolism through effects on lipid absorption and hepatic metabolism (Wu et al. 2007). However, the triglyceride response observed in the present study should be interpreted with caution because the experimental diets differed in ether extract concentration as a consequence of the formulation adjustments required to maintain isoenergetic diets. In particular, the lignocellulose diet contained a higher concentration of ether extract than the other treatments due to the greater inclusion of soybean oil. Therefore, the higher serum triglyceride concentration observed in this group cannot be attributed specifically to fiber fermentability, as differences in dietary lipid supply may also have contributed to this response. Thus, the present data do not allow the independent effects of fiber source and dietary fat concentration on serum triglycerides to be distinguished.

The transition period represents a critical window for mammary gland development and colostrum synthesis. Lobuloalveolar development intensifies during late gestation and continues into early lactation (Hurley, 2015), coinciding with colostrum production within the first 12–16 h postpartum (Theil et al. 2014). Nutritional strategies capable of modulating colostrum composition are therefore of great interest.

In the present study, colostrum fat and lactose concentrations were not affected by dietary fiber source, in agreement with previous studies (Loisel et al. 2013; Krogh et al. 2015; Li et al. 2021). Lactose concentration, in particular, is relatively stable and increases gradually during early lactation (Hurley 2015). Although SCFA have been proposed as potential lipid precursors for mammary synthesis, this mechanism was not directly evaluated in the present study, and no effect of dietary fiber source on colostrum fat concentration was observed, contrasting with findings reported by Zhou et al. (2020).

Conversely, colostrum protein concentration was significantly higher in sows fed micronized citrus pulp, a soluble and highly fermentable fiber source. Colostrum protein content largely reflects immunoglobulin concentration, which is critical for neonatal immunity (Klobasa et al. 1987; Martins et al. 2007). Although some studies report no effect of dietary fiber on colostrum protein (Quesnel et al. 2015), others have observed variations depending on fiber source and milk yield (Krogh et al. 2015). Differences among studies likely reflect variation in fiber physicochemical properties and their metabolic consequences.

Despite the positive effects on colostrum composition, colostrum yield was not affected by dietary fiber source, consistent with findings reported by Krogh et al. (2015). It is noteworthy, however, that sows nursing larger litters tend to produce more colostrum due to increased mammary stimulation immediately after farrowing (Grahofer and Plush 2023), which may partially explain variability across studies.

Overall, the present findings highlight that dietary fiber source influences reproductive and lactation outcomes, fecal consistency, and specific physiological responses in sows, including serum triglyceride and colostrum protein concentrations. Lignocellulose and micronized citrus pulp emerged as particularly effective functional fibers during late gestation, reinforcing their strategic importance in modern sow nutrition. Further research is warranted to elucidate the specific mechanisms through which different fiber fractions influence colostrogenesis and neonatal performance.

## Conclusion

Dietary fiber inclusion during late gestation improved reproductive and lactation outcomes in sows compared with a fiber-free control diet. Lignocellulose was particularly effective in reducing stillbirth rate and improving fecal score, while micronized citrus pulp increased colostrum protein concentration. Dietary treatments did not affect colostrum fat, lactose concentration, or yield. Overall, these findings indicate that the effects of dietary fiber depend on the fiber source and support its strategic use to improve specific reproductive and lactation outcomes in sows.

## Data Availability

The data that support the findings of this study are available on request from the corresponding author.
